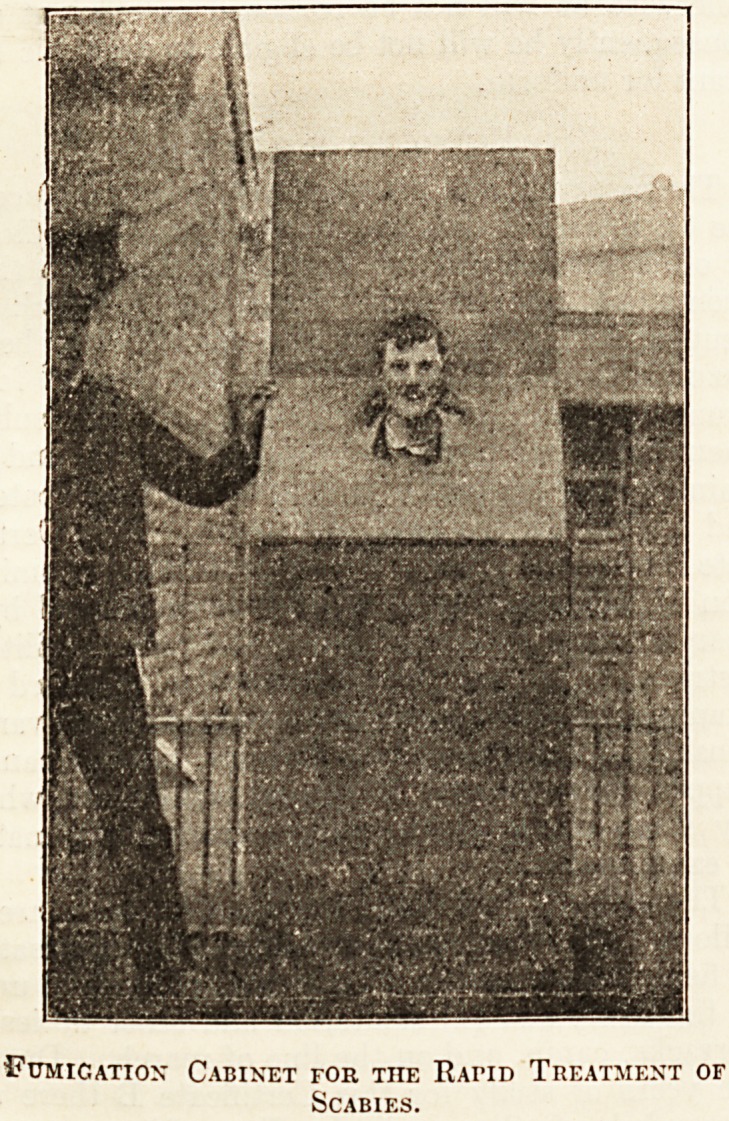# A Rapid Method of Curing Itch

**Published:** 1909-03-06

**Authors:** 


### THE GENERAL PRACTITIONER'S COLUMN.

[Contributions to this Column are invited, and if accepted will be paid for.]

### A RAPID METHOD OF CURING ITCH.

### THE SULPHUR FUMIGATION CABINET.

The accompanying photograph gives one a very good idea of a method of treating scabies that is very much in vogue in the workhouses of South Wales,

Fumigation Cabinet for the Rapid Treatment of Scabies.

such as Swansea, Carmarthen, and Haverfordwest. It is a method that is not described in many of the text-books, so I thought that a short description of it might be of interest.

It consists of an irregular-shaped box, with a door at the back through which the man enters; the upper part of the front is sloping, and has a hole in it large enough for a man to put his head through. At the left hand side is a conical-shaped flue, made of metal, in which is placed a sulphur candle.

The treatment consists in placing the man in the box with his head projecting through the hole, and his neck closely packed with cloths so that no fumes can pass up to his head. The door at the back is at the same time rendered fume-tight, and then the candle is lighted. The fumes ascend into the box, and, in addition to filling the box, also give rise to a considerable amount of heat, with the result that the pores of the skin are opened and the sulphur comes into closer contact with the acarus, and readily destroys the parasite. The sitting lasts for about three-quarters of an hour, and it is found that one sitting is generally sufficient for getting rid of the complaint.

It is a very simple method and much cleaner than the treatment by means of sulphur ointment. Another advantage that it possesses is that one can disinfect the clothes at the same time, a very necessary precaution against a recurrence. This method has worked for years, and no mishap has been recorded. At the Bridgend Cottage Homes it has been used for some time for the children with very happy results.

## Figures and Tables

**Figure f1:**